# Automated gait event detection for exoskeleton-assisted walking using a long short-term memory model with ground reaction force and heel marker data

**DOI:** 10.1371/journal.pone.0315186

**Published:** 2025-02-10

**Authors:** Xiaowen Chen, Anne E. Martin

**Affiliations:** Mechanical Engineering Department, Pennsylvania State University, University Park, Pennsylvania, United States of America; Ningbo University, CHINA

## Abstract

Traditional gait event detection methods for heel strike and toe-off utilize thresholding with ground reaction force (GRF) or kinematic data, while recent methods tend to use neural networks. However, when subjects’ walking behaviors are significantly altered by an assistive walking device, these detection methods tend to fail. Therefore, this paper introduces a new long short-term memory (LSTM)-based model for detecting gait events in subjects walking with a pair of custom ankle exoskeletons. This new model was developed by multiplying the weighted output of two LSTM models, one with GRF data as the input and one with heel marker height as input. The gait events were found using peak detection on the final model output. Compared to other machine learning algorithms, which use roughly 8:1 training-to-testing data ratio, this new model required only a 1:79 training-to-testing data ratio. The algorithm successfully detected over 98% of events within 16ms of manually identified events, which is greater than the 65% to 98% detection rate of previous LSTM algorithms. The high robustness and low training requirements of the model makes it an excellent tool for automated gait event detection for both exoskeleton-assisted and unassisted walking of healthy human subjects.

## 1. Introduction

Gait analysis is crucial for understanding human locomotion [1], diagnosing medical conditions [2], and designing personalized interventions [3]. When performing gait analysis, there are two key events in each stride for each leg: heel strike (HS), which is when the heel first contacts the ground and begins the stance phase, and toe off (TO), which is when the toe first leaves the ground and starts the swing phase. Accurate identification of HS and TO events is essential for segmenting walking data and analyzing temporal gait parameters [4]. Gait events are typically detected using ground reaction force (GRF) [5] or kinematic data [6]. Though these methods provide accurate and efficient gait event detection for healthy subjects, they can be unreliable when detecting gait events for subjects with disabilities, such as those with cerebral palsy [7–9]. Previous methods for gait event detection in subjects with cerebral palsy have used kinematic data with heuristics [7,8] or long short-term memory (LSTM) models [9]. These methods detected 66–93% of gait events within 16–20ms of the manually identified events [7,9]. Kinematic methods are also likely to be less reliable when subjects are wearing an assistive walking device, such as an exoskeleton, due to the altered walking pattern [10,11].

**Fig 1:**
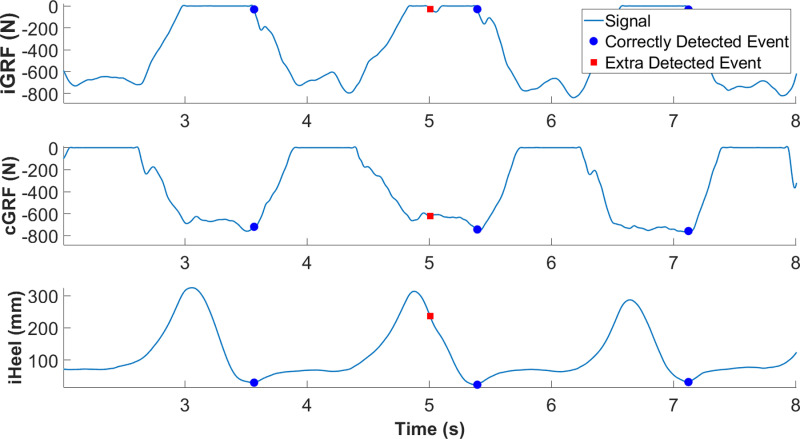
Example of a HS being wrongly detected by the GRF threshold method due to foot-dragging. The ipsilateral GRF (iGRF), contralateral GRF (cGRF), and ipsilateral heel height (iHeel) signals are shown. When the foot accidentally contacted the force plate, the signal crossed the threshold, causing an extra event to be detected.

Typically, when force plates [5] or pressure-sensitive insoles [12–14] are available, gait events are detected automatically using low-pass filtered GRF signals and an appropriate threshold. Using GRF as a direct indicator for gait events is intuitively aligned with their inherent definitions. While the event times can vary slightly depending on the filter cut-off frequency and force threshold [15], GRF are typically considered the gold standard for testing other gait event detection methods [4,6,16–18]. However, when subjects walk with exoskeletons, the device introduces significant modifications to their gait patterns [10]. The “clean” strikes (i.e., the entire foot lies on its own force plate during stance without touching down prematurely during swing) often assumed in GRF-based event detection methods [5,6] become less frequent. Subjects often drift side-to-side while walking, frequently resulting in a foot stepping on the wrong force plate. In addition, instances of “foot drag” (i.e., the foot makes premature ground contact) become a common occurrence (Fig 1).

When GRFs are unavailable, kinematic methods use motion capture marker data [4,6–8,16–20] or inertial measurement unit data [21,22] to detect events. Many of these methods use heuristics based on foot position and/or velocity. For healthy gait and some impaired gaits, these methods perform similarly to GRF-based methods, with average differences between 17 to 91ms [7]. However, these methods fail when gait significantly deviates from the typical pattern, as is typical with exoskeleton-assisted walking [10]. Recent work has started to use machine learning (ML) approaches with kinematic data for gait event detection [9,23–25]. These algorithms typically use multiple kinematic signals as inputs and generate Gaussian-distributed event prediction curves. LSTM algorithms are the most common, although other algorithms have also been used. However, these methods have primarily been designed for children with cerebral palsy and are generally not validated with GRF data. These ML algorithms still struggle to provide consistent results across diverse gait patterns and populations [9,24]. Therefore, it is unlikely that the algorithms would directly work for exoskeleton-assisted walking. Moreover, for exoskeleton-assisted walking, markers generally have to be positioned on the exoskeletons instead of directly on the participant’s skin, introducing additional differences between the original algorithm design and its use for exoskeleton experiments.

Therefore, relying solely on kinematic data or GRF signals and simple thresholds may not yield consistent and reliable event detection for exoskeleton-assisted walking. When the automatic detection methods fail, researchers must manually identify events, a very time-consuming process. Thus, alternative automated methods are needed. Because many exoskeleton experiments record both kinematic and GRF data, a method that utilizes both types of signals may perform better than a method that only uses one signal type.

As a new approach for advanced data analysis, ML, particularly deep learning, has shown potential in various applications, including gait analysis [9,23–28]. ML models learn complex patterns within the data, making them suitable for detecting gait events across different subjects and conditions. Because kinematic and GRF signals are time series, ML methods tailored to time series are likely to perform best. Hybrid convolutional neural network (CNN) and recurrent neural network (RNN) models have demonstrated promising results in predicting gait events using a single waist-worn wearable sensor [25]. However, both CNN and RNN can struggle to capture long-term dependencies. In contrast, LSTM, a specialized RNN, is adept at handling time series due to its inherent capability to capture and retain patterns within temporal sequences [29]. Given the cyclic and repetitive nature of human gait, LSTM is a promising tool for gait event detection. Thus, we chose to use an LSTM model for its ability to handle long-term dependencies in time series data. While it is probable that other time series methods could also work, testing a wide range of models was not within the scope of this study.

In this research, we propose an LSTM model to accurately identify gait events given both GRF and kinematic data for individuals using lower-limb exoskeletons. To the best of our knowledge, this is the first time kinematic and GRF data have been combined in a ML-based event detection algorithm. To develop the event detection model, two LSTM models were trained using a single signal (ipsilateral foot’s vertical GRF data or the ipsilateral heel marker height) and then combined into a final model. Data, along with manually identified gait events, from a total of 240 three-minute walking trials with and without exoskeletons were used to train and test the model. Three trials from two subjects containing approximately 700 HS events and 700 TO events were used for training, and the remaining 237 trials were used for testing. The model and manually identified gait events were compared, and a range of error metrics were used to evaluate the model’s performance. From these error metrices, we demonstrate that the algorithm can consistently detect events even when a subject’s foot is not cleanly on a single force plate and when a subject’s gait pattern differs from typical unassisted walking. This approach simplifies post-experiment data processing and reduces the dependence on expert knowledge for manual event detection.

## 2. Methods

All code used in this section is freely available from https://doi.org/10.26208/128M-YE61. This study was approved by the Penn State Institutional Review Board, STUDY00012828.

### 2.1 Data collection and preprocessing

In this study, a total of 14 participants (12 male, 2 female, age 22.6 ± 3.0 years, height 172 ± 11.3 cm, mass 70.1 ± 12.1 kg) with no known gait abnormalities or orthopedic issues were recruited from December 15, 2021, to August 2, 2022. Each provided signed informed consent, and the study was approved by the Penn State Institutional Review Board. Participants performed walking trials—one baseline trial without the exoskeletons and approximately 20 trials while wearing a bilateral pair of custom-built ankle exoskeletons [30,31]. Each trial lasted around three minutes, with varying exoskeleton control parameters.

**Fig 2:**
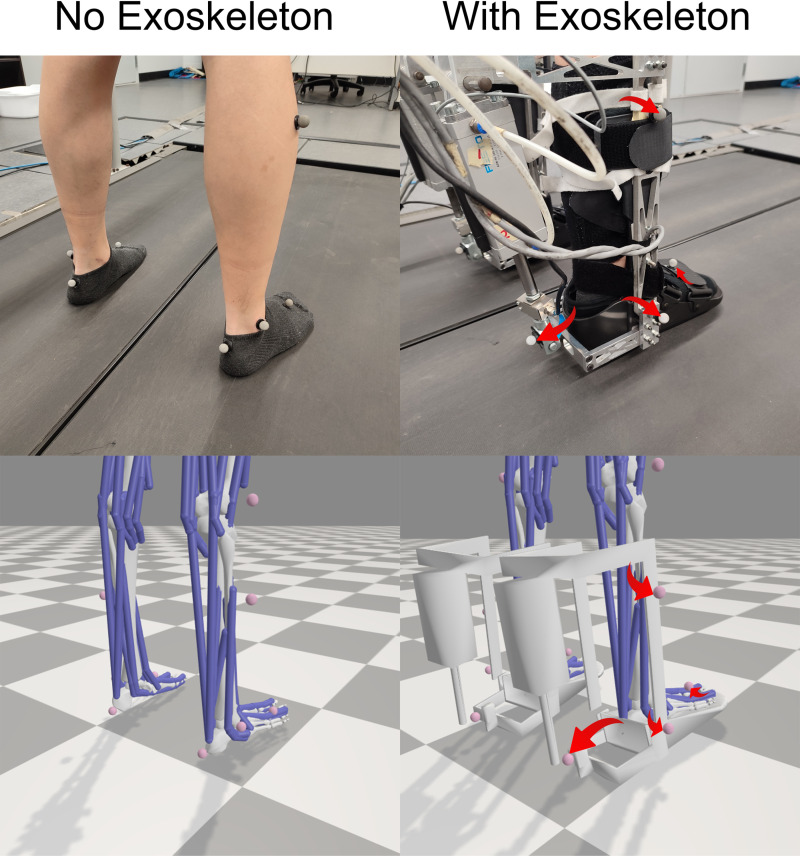
Relocated markers. The tibia, heel, and ankle markers are relocated onto the exoskeletons, while the toe markers are relocated onto the exoskeletons’ toe strap.

Before walking trials, each participant was outfitted with 16 reflective markers from the standard Plug-in Gait lower body marker set, placed on the hips, thighs, knees, tibias, ankles, heels, and toes [32]. During the no-exoskeleton trial, participants wore socks. For the subsequent 20 trials involving the exoskeletons, they wore the ankle exoskeletons. For the exoskeleton-assisted walking trials, markers on the tibiae, ankles, heels, and toes were relocated onto the exoskeletons (Fig 2).

All trials were conducted on a split-belt instrumented treadmill (Bertec, Columbus, OH) with the treadmill speed adjusted to a slow, height-normalized speed for each participant (0.491 ± 0.187 m/s). During data collection, fourteen cameras (Vicon, Oxford, UK) surrounding the treadmill recorded marker positions at 100 Hz. GRF data were collected at 1000 Hz using the force plates embedded in the treadmill.

Gaps in the marker data were filled using different methods depending on the number of frames per gap [32]. Gaps of less than 20 frames were filled using the cyclic method, while longer gaps were filled using a cubic spline or kinematic interpolation method. When a marker was missing at the beginning or end of a trial and the gap could not be filled, the trial was trimmed to omit the portion with the missing data. Moreover, trials with gaps that could not be filled were omitted from further analysis. After gap filling, a total of 240 trials (14 without the exoskeletons and 226 with the exoskeletons) with complete data were processed further. To reduce signal noise, GRF data was low pass filtered using a Butterworth zero-phase digital filter with a cut-off frequency of 20 Hz, while the marker data was smoothed with Woltring filter GCV mode and a smoothing parameter of 20 in Vicon Nexus [32]. To match the sampling rate of the GRF data, the marker position data was upsampled to 1000 Hz using the MATLAB “interpl” function.

Before developing the LSTM, gait events were initially found using the ipsilateral vertical GRF with a threshold of 1% of the subject’s weight. Manual adjustments were then performed to remove extra events, add in missing events, and correct inaccurately detected events. These errors occurred when the force plate was contacted by the contralateral foot or when foot dragging occurred.

**Fig 3:**
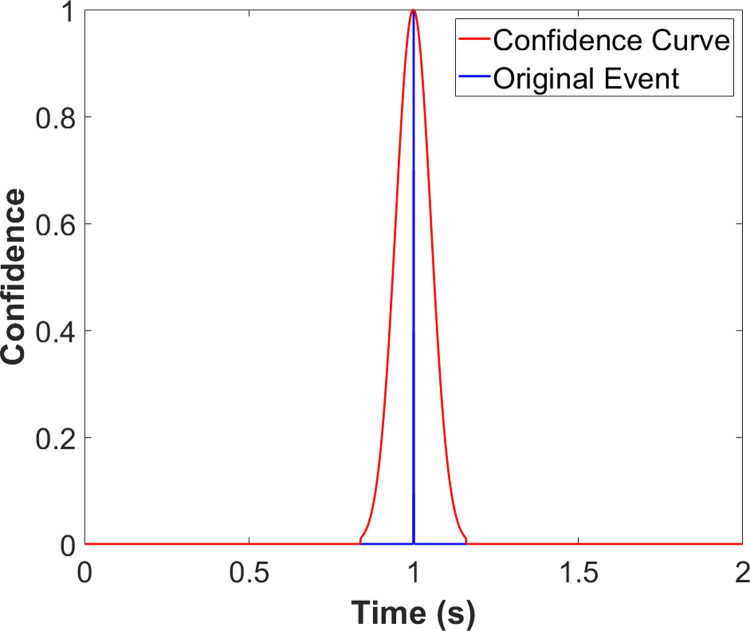
Sample plot of the confidence curve. The confidence curve peaks at each event and features a standard deviation of 53.3 ms.

For training and testing, the gait events were transformed into a Gaussian-distributed curve, termed the confidence curve (Fig 3). This curve had Gaussian distributions with a standard deviation of 53.3ms and a peak of 1 centered on each event. Segments between the Gaussian distributions were 0. For each trial, a total of four event curves were found, one each for left foot HS, right foot HS, left foot TO, and right foot TO.

### 2.2 LSTM model development

Single-layer LSTM neural networks (NN) were used for this study. For each NN, the LSTM layer was connected to a sequence input layer, a fully connected layer, and a regression layer. The model was trained in MATLAB using the “trainNetwork” function. The NN’s input was the ipsilateral foot’s vertical GRF data, the contralateral foot’s vertical GRF data, and/or the ipsilateral heel marker height. The output of the NN was the ipsilateral HS and TO confidence curves, where events were detected at peaks in the confidence curves. Training and testing were executed on a Quadro RTX 4000 with 8GB of VRAM (Nvidia, Santa Clara, CA).

#### 2.2.1 Hyperparameter tuning.

An iterative search was performed to identify optimal training hyperparameters. Because the hidden units in the LSTM represent the dimensionality of the input data, approximately 500 hidden units were needed to effectively capture 0.5 seconds, or approximately 1 step, of walking data in the model’s time series analysis. Thus, the number of hidden units were varied from 10–630. Given the limitation of available computational vRAM, a minibatch size of 200 was chosen as the maximum to balance computational efficiency with model performance. Therefore, the search varied the minibatch size from 10–200. The maximum number of epochs was kept constant at 100. For the hyperparameter tuning portion of the study, all three inputs (both GRF and the ipsilateral heel marker height) were used in a single LSTM model. For all hyperparameter configurations, the model was trained using a specific trial and then tested on all of that subject’s trials. The performance of each configuration was evaluated using the correlation coefficient between the LSTM and manual event confidence curves and the configuration with the highest correlation coefficient was chosen. The final parameters were a minibatch size of 110, 540 hidden units, and 500 epochs.

#### 2.2.2 Final model development.

Further testing of the model suggested that the three inputs unevenly contributed to the output. Therefore, to improve the performance, a weighed model output technique was applied. Separate LSTM networks were individually trained for each of the three inputs. The final confidence curve was calculated using a weighted formula:


$$C=Sign\left[C_{iGRF}\right]\cdot C_{iGRF}^{a}\cdot Sign\left[C_{cGRF}\right]\;\cdot \;C_{cGRF}^{b}\cdot Sign\left[C_{iHeel}\right]\;\cdot C_{iHeel}^{c},$$ (1)

where $C_{iGRF}$, $C_{cGRF}$, and $C_{iHeel}$ are the confidence curves generated by the three models trained using ipsilateral foot GRF, contralateral foot GRF, and ipsilateral heel marker data. The coefficients *a*, *b*, and *c* are the exponential weights for each individual model. Optimal weighting coefficients (a=0.3, b=0, c=0.01) were determined through iterative testing on a single subject’s full trial set. The coefficients were varied between 0 and 3. The performance of each coefficient set was assessed by calculating the root mean square error (RMSE) of the final model events against the manual events. Since the weighting coefficient for C_cGRF_ model was 0, the C_cGRF_ terms were replaced by 1. Therefore, the final equation was


$$C=Sign\left[C_{iGRF}\right]\cdot C_{iGRF}^{0.3}\cdot Sign\left[C_{iHeel}\right]\cdot C_{iHeel}^{0.01}.$$ (2)

The LSTM models were trained incrementally with a decaying learning rate of 50% for every 50 epochs, utilizing three trials from two subjects. This corresponded to approximately 540,000 data points, with approximately 700 HS events and 700 TO events. The three trials included a no-exoskeleton trial and two exoskeleton-assisted trials. Moreover, the chosen exoskeleton-assisted trials had both typical and uncommon gait patterns. To perform the incremental training, the left side of the no-exoskeleton trial was used first, followed by the right side of the no-exoskeleton trial, then the left side of the first exoskeleton trial, then the right side of the first exoskeleton trial, and so on.

**Fig 4:**
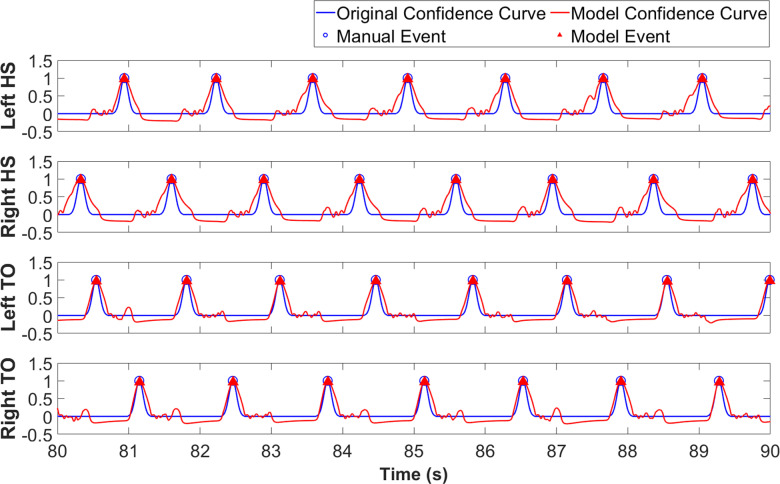
Representative model confidence output for a no-exoskeleton testing trial. LSTM detection curves peak at roughly the same time as the manual event confidence curves. LSTM HS detection curves show fluctuations before the peaks while the TO detection curves show fluctuations after the peaks.

The LSTM output confidence curves for HS and TO ranged from approximately 0 to 1, and the confidence curves peaked at each event (Fig 4). From these curves, gait events were identified using peak detection, with a minimum peak height of 0.3 and a minimum interval between peaks of 1000ms. This duration was chosen based on the walking speed and standard human gait patterns when wearing the exoskeletons.

### 2.3 Analysis

The 237 trials not used for training were used for testing. This corresponds to approximately 710 minutes of walking and contains approximately 56,000 of each event type. The testing data also included 12 subjects that the model had never seen. Model detected events were compared against the manual events. The model’s efficacy was evaluated using error metrics including mean error (ME), median absolute error (MAE), interquartile range of the error (IQR), standard deviation of the error (SD), maximum error (MAX), successful detection rate (SR), and overall detection rate (DR). Successful detection was defined as a model-detected event that fell within 16ms of a manual event, which is the 84% coverage window of inter-raters variance [19]. The overall detection rate was the percentage of model events within 1000ms of a manual event. A positive error value indicated that the manual event was earlier. All error metrics were calculated by finding the closest model detected event to each manual event. The only exception was the SD, which was calculated using the successfully detected events only because otherwise a few large errors artificially increased the value. The goal was a SR above 95% and a DR exceeding 99.9% across all trials.

To quantify how much the combined model improved event detection, the ipsilateral GRF only, heel marker only, and final models were compared using the above error metrics plus the number of extra events. The extra events were calculated using the difference between the number of model and manual events, where a positive value indicates that model found extra events and a negative value indicates that the model missed events. When counting the number of events, the first model event was the closest model event to the first manual event and the last model event was the closest model event to the last manual event.

## 3. Results

### 3.1. Gait event detection accuracy

**Fig 5:**
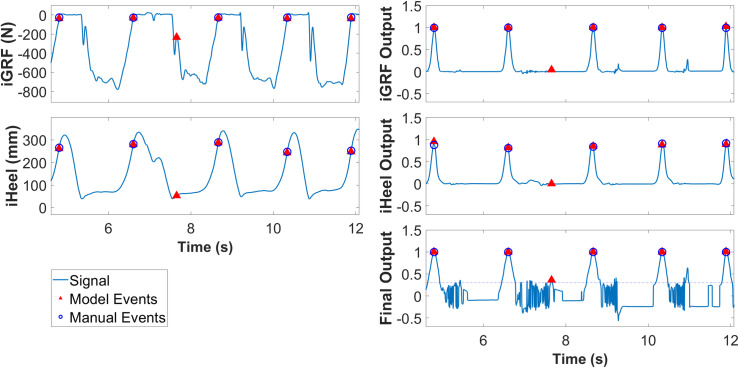
Signals around one of the extra events detected by the final model. Though both individual models’ outputs are low at the extra event, combining the two output signals amplified the noise, causing the final output to exceed the event threshold limit.

From the 240 trials, the LSTM model detected 56,618 HS and 56,620 TO events, while the manual identification method identified 56,618 HS and TO events each. Thus, the model found two extra TO events, from a total of 113,236 events. The extra events were caused by noise in the final model output exceeding the event threshold limit (Fig 5).

The event locations were very similar between the two methods, with 97.9% of HS and 99.9% of TO events detected within 16 ms of the manual events (Table 1). Out of all the events, only 1126 HS and 28 TO events had differences greater than 16ms between LSTM and manual events. The model achieved a perfect detection rate of 100%.

**Table 1:** Summary metrics for differences between LSTM and manual events for all trials from all subjects. All error metrics were extremely small for both HS and TO. However, the TO detection was more precise but less accurate than HS detection.

	ME (ms)	MAE (ms)	IQR (ms)	SD (ms)	MAX (ms)	SR (%)	DR (%)
HS	−0.8301	3	4	4.554	497	97.937	100
TO	−2.869	3	1	1.664	480	99.871	100

The metrics include mean error (ME), median absolute error (MAE), interquartile range of the error (IQR), standard deviation of the error (SD), maximum absolute error (MAX), success rate (SR), and detection rate (DR).

**Fig 6:**
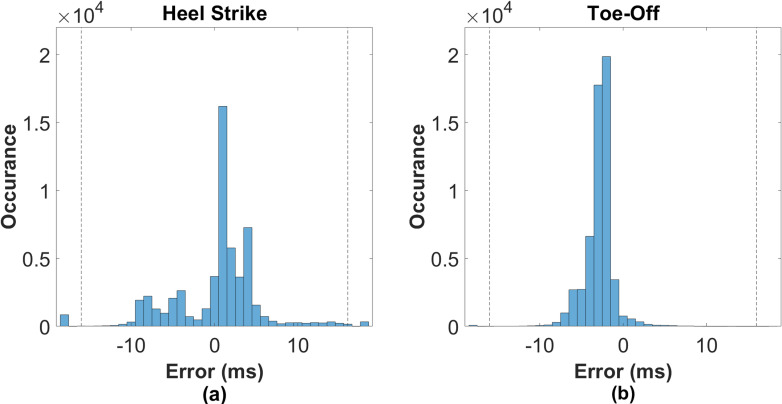
Difference between LSTM and manual (a) heel strikes and (b) toe offs for all trials. The positive values indicating the manual event were earlier. Differences greater than ± 16ms are clipped and shown outside the dotted lines. HS differences are well-centered around zero but exhibit moderate spread. TO differences are shifted slightly left with little spread.

The two methods identified HS at essentially the same time on average, with a signed mean error of less than 1ms (Fig 6a). Except for the maximum error, all error metrics were less than 5ms. As expected, the maximum error was substantially higher. The model TO were slightly earlier on average (−2.869ms) than the manual events (Fig 6b). However, compared to HS, the TO detection showed higher performance, with lower IQR, MAX, and SD, and higher SR.

### 3.2 Error metrics across training and testing data sets

The LSTM model performed consistently across training and testing datasets. The model showed similar performance between the 3 training trials and the 237 testing trials (Table 2). When split by subject, the performance for the 2 training subjects was very slightly better than for the 12 testing subjects.

**Table 2:** Neural network error metrics for training and testing data sets. The neural network performs similarly between training and testing trial sets and performs slightly better for training subjects compared to testing subjects.

	ME (ms)	MAE (ms)	IQR (ms)	SD (ms)	MAX (ms)	SR (%)
By Trials						
Training HS	−3.03	2	5	4.54	445	98.36
Testing HS	−0.804	3	4	4.55	497	97.93
Training TO	−2.29	2	1	1.76	7	100
Testing TO	−2.88	3	1	1.66	480	99.87
By Subjects						
Training HS	−0.860	2	2	3.90	445	98.09
Testing HS	−0.824	3	5	4.67	497	97.91
Training TO	−2.42	2	1	1.17	480	99.95
Testing TO	−2.96	3	2	1.74	205	99.86

The metrics include mean error (ME), median absolute error (MAE), interquartile range of the error (IQR), standard deviation of the error (SD), maximum absolute error (MAX), success rate (SR), and detection rate (DR).

### 3.3 Comparison with model components

Compared to the two individual models comprising the final model, the final model had lower maximum errors (Table 3). The heel marker model had significantly higher error metrics and lower SR compared to the other two models. Except for the maximum error, the GRF model had similar or slightly lower error metrics compared to the final model. However, the final model had excellent error metrics, showing a balanced performance in terms of both accuracy and precision.

**Table 3:** Comparison between individual and final models. The GRF and heel model are the models trained using only the ipsilateral GRF signal and only the ipsilateral heel height signal, respectively. The heel marker model performs significantly worse than the other two models. The GRF and final models had similar error metrics.

Model	Event Type	Statistics							Extra Events
		ME	MAE	RMSE	SD	MAX	SR	DR	
GRF Model	HS	−0.72	3	4	4.53	1714	98.01	100	−3
	TO	−3.0	3	1	1.47	480	99.95	100	0
Heel Model	HS	−36	29	77	9.44	4065	29.38	99.86	−71
	TO	6.4	21	40	9.18	2082	41.45	99.96	−24
Final Model	HS	−0.8	3	4	4.55	497	97.94	100	0
	TO	−2.9	3	1	1.66	480	99.87	100	2

The metrics include mean error (ME), median absolute error (MAE), interquartile range of the error (IQR), standard deviation of the error (SD), maximum absolute error (MAX), success rate (SR), detection rate (DR), and extra events.

## 4. Discussions and conclusions

This study introduced a novel approach to gait event detection by combining kinematic and GRF LSTM models, addressing inherent limitations in existing GRF, kinematic, and machine learning methodologies. Moreover, compared to existing methods, the new method enhanced the reliability of gait event detection for subjects with atypical walking behavior introduced by a pair of ankle exoskeletons. The LSTM model developed in this work demonstrated high precision in gait event detection, successfully identifying over 98% of HS and TO events within 16 ms of manual events.

The presented algorithm detected events at higher rates than existing methods (Table 4). When using kinematics with heuristic methods, most algorithms find 95% of HS within 33ms and 60−90% of TO within 33ms for children with cerebral palsy [19]. Previous work using neural networks and healthy adults walking normally are more successful, with one study detecting 98% of events within 20ms [25] and another detecting 99% of HS and 95% of TO events [24]. One previous study that used an LSTM to detect gait events in children with cerebral palsy did not perform well, with only roughly 80% of HS and 65% of TO events detected within 16ms of the manually identified events [9]. Although the current study investigated a different population walking with ankle exoskeletons, the presented LSTM method using both GRF and kinematics detected 98% of HS and almost 100% of TO within 16ms, surpassing the performance of any previously mentioned algorithm. Moreover, compared to conventional event detection using the GRF signal with a threshold of 1% of a subject’s weight, significantly fewer extra events were detected by the machine learning algorithm. In the analysis of 113,236 actual events in this study, the conventional GRF threshold method identified 13,640 extra events. In contrast, the final model utilizing LSTM only detected two extra events, a much lower ratio of fake detections.

**Table 4:** Matrix of comparison of the proposed method with other methods. The new method shows significantly lower ME, MAE, and STD, and the highest SR and DR among all methods shown in this table. Moreover, the percentage of extra events detected from the new method is particularly low.

Study	Method	Population	Event Type	Input Signal	ME	MAE	STD	SR/Range	DR	Extra Events
[9]	LSTM	CCP	HS	KINE				82.5% /16ms	90%	18%
			TO					66.2% /16ms	72%	34%
[24]	LSTM	ADCP	HS	KINE	18				99%	
			TO		13				95%	
[6]	H	HA	HS	KINE	16		15			
			TO		9		15			
[6]	H	CGP	HS	KINE	−3		9			
			TO		−6		26			
[16]	H	HA	HS	KINE	1					
			TO							
[7]	H	HA	HS	KINE	1	12				
			TO		−2	11				
[7]	H	CCP	HS	KINE	27	32				
			TO		−14	18				
[19]	H	CP	HS	KINE				95% /33ms		
			TO					60–90% /33ms		
[25]	NNs	OA	HS	IMU				98% /20ms		
			TO							
Cur	Weighed LSTM	HAE	HS	KINE & GRF	−1	3	5	97.9% /16ms	100%	0%
			TO		−3	3	2	99.9% /16ms	100%	4e–5%
Cur	T	HAE	HS	GRF						12%
			TO							

Cur: current study, H: Heuristics, LSTM: Long Short-Term Memory, NNs: other kinds of neural networks, LSTM w/weight: the proposed method in this paper of combined weighed LSTMs, T: threshold, CCP: Children with cerebral palsy, ADCP: adults with disorders or cerebral palsy, HA: healthy adults, CGP: Children with gait problems, OA: old adults, HAE: healthy adults with or without exoskeletons, KINE: motion captured kinematics data, IMU: inertial measurement unit data, and GRF: ground reaction force. The metrics include mean error (ME) in ms, median absolute error (MAE) in ms, standard deviation of the error (SD) in ms, success rate (SR) in percentage and their respective range of limit (Range) in ms, detection rate (DR), and extra events in percentage of total detected events.

Most of the heuristics kinematic methods detect gait events with a mean error of 10−30ms compared to GRF detected events [19]. Depending on the LSTM method used, the trimmed mean error ranged from 5ms to 20ms in previous work [9,24]. In comparison, the error for the presented LSTM method was substantially lower, with mean errors of less than 3ms. From a practical standpoint, this error is negligible because it is within one frame of typical motion capture sampling rates.

Compared to other machine learning algorithms, this new method required a significantly smaller training data set. Two previous LSTM-based methods with kinematic input data had a roughly 8:1 training-to-testing data ratio [9,24], while the current method required a much lower 1:79 training-to-testing data ratio. The reduced ratio suggests that this algorithm is particularly easy to implement and requires much less manually labeled data, significantly reducing the time required to identify gait events. Thus, compared to other automatic gait event detection methods, the time and effort required to implement this new algorithm is relatively low while achieving very high accuracy.

Part of the reason for the low training-to-testing data ratio was careful selection of the training data sets. Before the training began, we observed that three distinct types of foot strikes exist throughout the walking trials. They were foot dragging (premature foot contact with force plate), unclean foot strikes (foot landing on both left and right force plates), and clean foot strikes (foot landing entirely on a single force plate). The three training trials were selected so that the training data included each of these behaviors, ensuring the model was exposed to all types of foot strike conditions. We also found that performing the initial training with a relatively clean no-exoskeleton trial followed by messier exoskeleton trials gave the best results.

The final model’s performance did not substantially surpass that of the GRF model alone, showing the dominant effect of the ground reaction force. We assumed that including the kinematics would increase the reliability of the gait event detections. Because the GRF model relied solely on the GRF signal, when subjects stepped on both force plates, the distortion of the GRF signal could cause failure in detection and introduce extra or missing events. By using multiple signals, we expected that the final model would be better able to successfully detect events. However, it appears that with an appropriate training regime, the GRF model alone was able to handle even very messy input signals, making the kinematic model redundant. Similar to previous work [9,24], the kinematic-only model was not able to detect all events. Given that the GRF model could, this suggests that kinematic data may not be useful and may actually be harmful when GRF data is available for gait event detection. This may partially explain why initial attempts to create a single LSTM model using GRF and kinematic data were not as successful as desired.

Despite its robust performance, the model exhibits certain limitations. The model is suited for post-experimental data analysis rather than real-time event detection. The model was only validated using unassisted and ankle exoskeleton-assisted walking for young healthy subjects. It is possible that the performance could be different in other populations, particularly if the models are not retrained. While it did not substantially affect the results, the final output curve is extremely noisy (Fig 5). The noise originates primarily from variations in the input data causing small fluctuations in the individual LSTM model output. Although these fluctuations are minor, they can be significantly amplified in the final model due to the configuration of Eq. 2. This occasionally caused extra events to be identified. Refining the weighting algorithm could further enhance the final output’s stability and precision. Moreover, adding anterior-posterior GRF signal to the input might also improve the performance.

In conclusion, the presented LSTM model is a reliable instrument for gait event detection, with all true events and very few extra events detected. The model’s events were at very similar times to manually identified events, with over 98% of events within 16ms and mean differences of less than 3ms. Its performance was consistent across 235 trials and only three required training trials, suggesting it is highly generalizable.
